# Supplementary material for: Cognition in older adults in Uganda: Correlates, trends over time and association with mortality in prospective population study
Source: PLOS Glob Public Health. 2023 Nov 3;3(11):e0001798. doi: 10.1371/journal.pgph.0001798 (PMC10624290; doi:10.1371/journal.pgph.0001798)
Supplement: S1 Table — (DOCX) [file pgph.0001798.s001.docx]

S1 Table. Detailed baseline characteristics by wave and sex

|  |  | **Wave 1** | | **Wave 2** | | **Wave 3** | |
| --- | --- | --- | --- | --- | --- | --- | --- |
|  |  | **Men** | **Women** | **Men** | **Women** | **Men** | **Women** |
|  |  | **n (%)** | **n (%)** | **n (%)** | **n (%)** | **n (%)** | **n (%)** |
|  |  |  |  |  |  |  |  |
| **Residence** | Rural | 106 (54) | 150 (48) | 1 (2) | 7 (7) | 55 (69) | 58 (60) |
|  | Urban | 92 (47) | 161 (52) | 44 (98) | 74 (9) | 25 (31) | 38 (40) |
|  |  |  |  |  |  |  |  |
| **Age group** | 50-59 | 70 (35) | 108 (35) | 27 (60) | 53 (65) | 41 (51) | 40 (42) |
|  | 60-69 | 51 (26) | 99 (32) | 15 (33) | 20 (25) | 26 (33) | 31 (32) |
|  | 70-79 | 54 (27) | 73 (24) | 3 (7) | 6 (7) | 6 (8) | 18 (19) |
|  | 80+ | 23 (12) | 31 (10) | 0 (0) | 2 (3) | 7 (9) | 7 (7) |
|  |  |  |  |  |  |  |  |
| **Education** | No formal education | 29 (15) | 89 (29) | 2 (4) | 13 (16) | 11 (14) | 24 (25) |
|  | Less than primary | 82 (41) | 157 (51) | 22 (49) | 29 (36) | 38 (48) | 44 (46) |
|  | Completed primary school | 26 (13) | 27 (9) | 9 (20) | 20 (25) | 17 (21) | 10 (10) |
|  | More than primary | 61 (31) | 37 (12) | 12 (27) | 19 (24) | 14 (18) | 18 (19) |
|  |  |  |  |  |  |  |  |
| **Marital status** | Married/cohabiting | 119 (60) | 46 (15) | 29 (64) | 11 (14) | 58 (73) | 22 (23) |
|  | Divorced/separated/never married | 40 (20) | 65 (21) | 13 (29) | 21 (26) | 14 (18) | 29 (31) |
|  | Widowed | 39 (20) | 200 (64) | 3 (7) | 49 (61) | 8 (10) | 44 (46) |
|  |  |  |  |  |  |  |  |
| **Socio-economic position** | 1 (Lowest) | 48 (25) | 70 (23) | 5 (11) | 16 (20) | 12 (15) | 22 (23) |
|  | 2 | 41 (21) | 69 (23) | 11 (24) | 21 (26) | 17 (22) | 14 (15) |
|  | 3 | 33 (17) | 65 (21) | 17 (38) | 19 (24) | 21 (27) | 18 (19) |
|  | 4 | 43 (22) | 62 (20) | 11 (24) | 18 (22) | 13 (17) | 23 (25) |
|  | 5 (Highest) | 31 (16) | 39 (13) | 1 (2) | 6 (8) | 16 (20) | 17 (18) |
|  |  |  |  |  |  |  |  |
| **Tobacco use** | Never used tobacco | 89 (45) | 253 (82) | 25 (56) | 67 (83) | 39 (49) | 79 (82) |
|  | Current tobacco use | 52 (26) | 37 (12) | 5 (11) | 6 (7) | 17 (21) | 11 (12) |
|  | Previous tobacco use | 57 (29) | 20 (7) | 15 (33) | 8 (10) | 24 (30) | 6 (6) |
|  |  |  |  |  |  |  |  |
| **Alcohol use** | No | 37 (19) | 89 (29) | 4 (9) | 28 (35) | 22 (28) | 37 (39) |
|  | Yes | 161 (81) | 221 (71) | 41 (91) | 53 (65) | 58 (73) | 59 (62) |
|  |  |  |  |  |  |  |  |
| **BMI (kg/m^2^)** | <18.5 | 36 (18) | 35 (11) | 11 (24) | 11 (14) | 14 (18) | 8 (8) |
|  | 18.5- | 133 (67) | 161 (52) | 30 (67) | 49 (61) | 58 (73) | 55 (57) |
|  | 25- | 19 (10) | 66 (21) | 4 (9) | 12 (15) | 7 (9) | 19 (20) |
|  | 30+ | 10 (5) | 49 (16) | 0 (0) | 9 (11) | 1 (1) | 14 (15) |
|  |  |  |  |  |  |  |  |
| **Hypertension^1^** | No hypertension | 114 (58) | 153 (49) | 30 (67) | 48 (59) | 51 (64) | 49 (51) |
|  | Hypertension | 84 (42) | 158 (51) | 15 (33) | 33 (41) | 29 (36) | 47 (49) |
|  |  |  |  |  |  |  |  |
| **Diabetes^2^** | No | 191 (97) | 290 (94) | 45 (100) | 79 (98) | 77 (96) | 89 (93) |
|  | Yes | 7 (4) | 19 (6) | 0 (0) | 2 (3) | 3 (4) | 7 (7) |
|  |  |  |  |  |  |  |  |
| **Stroke^3^** | No | 188 (95) | 291 (94) | 42 (93) | 79 (98) | 79 (99) | 92 (96) |
|  | Yes | 10 (5) | 20 (6) | 3 (7) | 2 (3) | 1 (1) | 4 (4) |
|  |  |  |  |  |  |  |  |
| **Angina^3^** | No angina | 148 (75) | 230 (74) | 36 (80) | 62 (77) | 67 (84) | 81 (84) |
|  | Angina | 49 (25) | 81 (26) | 9 (20) | 19 (24) | 13 (16) | 15 (16) |
|  |  |  |  |  |  |  |  |
| **HIV** | HIV negative | 113 (57) | 197 (63) | 2 (4) | 11 (14) | 0 | 0 |
|  | HIV positive | 85 (43) | 114 (37) | 43 (96) | 70 (86) | 79 (100) | 92 (100) |
| 1. Defined as systolic blood pressure≥140, diastolic blood pressure≥90, or self-report of use of antihypertensive medication 2. Defined as clinician diagnosis of diabetes 3. Defined as clinician diagnosis or a suggestive history (hemisensory loss or hemiparesis lasting >24 hours for stroke; chest pain on exertion for angina) | | | | | | | |
