# Supplementary material for: Cognition in older adults in Uganda: Correlates, trends over time and association with mortality in prospective population study
Source: PLOS Glob Public Health. 2023 Nov 3;3(11):e0001798. doi: 10.1371/journal.pgph.0001798 (PMC10624290; doi:10.1371/journal.pgph.0001798)
Supplement: S2 Table — (DOCX) [file pgph.0001798.s002.docx]

S2 Table. Mean score and correlates of each cognition component

|  | | **N** | **Recall^1^** | | | **Digit span^2,3^** | | | **Verbal fluency^3^** | | |
| --- | --- | --- | --- | --- | --- | --- | --- | --- | --- | --- | --- |
|  |  |  | **Mean cognition score^4^** | **Linear regression coefficient^5^**  **(95% CI)** | **p-value** | **Mean cognition score^4^** | **Linear regression coefficient^5^ (95% CI)** | **p-value** | **Mean cognition score^4^** | **Linear regression coefficient^5^ (95% CI)** | **p-value** |
|  | | | | | |  | | |  | | |
| **Wave at recruitment** | 1 | 509 | 0.18 | Ref | <0.0001 | 0.008 | Ref | 0.003 | 0.05 | Ref | <0.0001 |
|  | 2 | 126 | -0.27 | -0.74 (-0.94;-0.54) |  | 0.006 | -0.34 (-0.53;-0.14) |  | -0.29 | -0.49 (-0.69;-0.29) |  |
|  | 3 | 176 | -0.17 | -0.46 (-0.62;-0.30) |  | 0.06 | -0.05 (-0.21;0.11) |  | 0.31 | 0.15 (-0.02;0.31) |  |
|  | | | | | |  | | |  | | |
| **Residence** | Urban | 377 | -0.03 | Ref | \| 0.30 \| \| --- \| \|  \| | -0.12 | Ref | \| 0.005 \| \| --- \| \|  \| | 0.25 | Ref | \| 0.55 \| \| --- \| \|  \| |
|  | Rural | 434 | 0.09 | 0.07 (-0.07;0.22) |  | 0.14 | 0.20 (0.06;0.34) |  | -0.07 | -0.04 (-0.19;0.10) |  |
|  | | | | | |  | | |  | | |
| **Sex** | Male | 323 | -0.07 | Ref | \| 0.02 \| \| --- \| \|  \| | 0.17 | Ref | \| 0.04 \| \| --- \| \|  \| | 0.11 | Ref | \| 0.004 \| \| --- \| \|  \| |
|  | Female | 488 | 0.10 | 0.19 (0.03;0.36) |  | -0.08 | -0.17 (-0.34;-0.01) |  | 0.009 | -0.24 (-0.41;-0.07) |  |
|  | | | | | |  | | |  | | |
| **Age group** | 50-59 | 351 | 0.27 | Ref | <0.0001 | 0.30 | Ref | <0.0001 | 0.19 | Ref | <0.0001 |
|  | 60-69 | 269 | 0.04 | -0.26 (-0.41;-0.11) |  | -0.04 | -0.32 (-0.48;-0.17) |  | 0.02 | -0.14 (-0.29;0.02) |  |
|  | 70-79 | 179 | -0.29 | -0.66 (-0.84;-0.48) |  | -0.25 | -0.46 (-0.64;-0.28) |  | -0.16 | -0.31 (-0.49;-0.13) |  |
|  | 80+ | 79 | -0.69 | -1.03 (-1.27;-0.79) |  | -0.63 | -0.78 (-1.03;-0.54) |  | -0.54 | -0.69 (-0.94;-0.44) |  |
|  | | | | | |  | | |  | | |
| **Education** | No formal education | 179 | -0.25 | Ref | \| 0.08 \| \| --- \| \|  \| | -0.63 | Ref | \| <0.0001 \| \| --- \| \|  \| | -0.35 | Ref | \| 0.005 \| \| --- \| \|  \| |
|  | Any formal education | 698 | 0.06 | 0.15 (-0.02;0.32) |  | 0.16 | 0.56 (0.39;0.73) |  | 0.08 | 0.24 (0.07;0.41) |  |
|  | | | | | |  | | |  | | |
| **Marital status** | Married | 318 | 0.01 | Ref | \| 0.69 \| \| --- \| \|  \| | 0.19 | Ref | \| 0.35 \| \| --- \| \|  \| | 0.16 | Ref | \| 0.71 \| \| --- \| \|  \| |
|  | Not married | 559 | -0.004 | -0.03 (-0.19;0.13) |  | -0.11 | -0.07 (-0.23;0.08) |  | -0.09 | -0.03 (-0.19;0.13) |  |
|  | | | | | |  | | |  | | |
| **SEP** | 1 (poorest) | 173 | -0.31 | Ref | <0.0001 | -0.37 | Ref | 0.002 | -0.14 | Ref | 0.14 |
|  | 2 | 173 | 0.04 | 0.38 (0.19;0.57) |  | 0.001 | 0.31 (0.12;0.51) |  | -0.04 | 0.11 (-0.09;0.30) |  |
|  | 3 | 173 | 0.001 | 0.26 (0.06;0.46) |  | 0.05 | 0.22 (0.02;0.42) |  | 0.14 | 0.2 (-0.01;0.4) |  |
|  | 4 | 173 | 0.21 | 0.42 (0.22;0.63) |  | 0.27 | 0.4 (0.19;0.6) |  | 0.12 | 0.2 (-0.01;0.41) |  |
|  | 5 (richest) | 174 | 0.05 | 0.56 (0.32;0.80) |  | 0.06 | 0.33 (0.09;0.57) |  | -0.05 | 0.29 (0.05;0.53) |  |
|  | | | | | |  | | |  | | |
| **Tobacco use** | Current | 135 | -0.14 | Ref | 0.66 | -0.16 | Ref | 0.98 | -0.07 | Ref | 0.86 |
|  | Previous | 135 | -0.05 | -0.03 (-0.26;0.20) |  | 0.02 | -0.01 (-0.23;0.22) |  | 0.11 | 0.06 (-0.17;0.30) |  |
|  | Never | 607 | 0.04 | -0.08 (-0.27;0.11) |  | 0.03 | 0.01 (-0.18;0.2) |  | -0.006 | 0.02 (-0.17;0.22) |  |
|  | | | | | |  | | |  | | |
| **Alcohol** | No alcohol | 634 | 0.001 | Ref | \| 0.99 \| \| --- \| \|  \| | 0.008 | Ref | \| 0.31 \| \| --- \| \|  \| | -0.008 | Ref | \| 0.24 \| \| --- \| \|  \| |
|  | Alcohol | 243 | 0.004 | 0.06 (-0.09;0.21) |  | -0.012 | 0.08 (-0.07;0.23) |  | 0.03 | 0.09 (-0.06;0.24) |  |
|  | | | | | |  | | |  | | |
| **BMI** | <18.5 | 126 | -0.38 | -0.27 (-0.46;-0.08) | 0.08 | -0.26 | -0.13 (-0.32;0.06) | 0.16 | -0.22 | -0.13 (-0.32;0.07) | 0.44 |
|  | 18.5- | 525 | 0.01 | Ref |  | 0.04 | Ref |  | 0.047 | Ref |  |
|  | 25- | 141 | 0.27 | 0.12 (-0.06;0.31) |  | 0.17 | 0.08 (-0.11;0.26) |  | 0.07 | 0.01 (-0.18;0.20) |  |
|  | 30+ | 86 | 0.03 | -0.1 (-0.33;0.13) |  | -0.17 | -0.16 (-0.38;0.07) |  | -0.08 | -0.12 (-0.35;0.11) |  |
|  | | | | | |  | | |  | | |
| **HTN** | No hypertension | 485 | -0.02 | Ref | \| 0.73 \| \| --- \| \|  \| | 0.02 | Ref | \| 0.89 \| \| --- \| \|  \| | -0.014 | Ref | \| 0.32 \| \| --- \| \|  \| |
|  | Hyper-tension | 393 | 0.02 | 0.02 (-0.11;0.16) |  | -0.03 | -0.01 (-0.14;0.13) |  | 0.018 | 0.07 (-0.07;0.21) |  |
|  | | | | | |  | | |  | | |
| **Diabetes** | No diabetes | 834 | -0.01 | Ref | \| 0.88 \| \| --- \| \|  \| | -0.01 | Ref | \| 0.82 \| \| --- \| \|  \| | -0.007 | Ref | \| 0.73 \| \| --- \| \|  \| |
|  | Diabetes | 41 | 0.27 | 0.02 (-0.29;0.34) |  | 0.26 | -0.04 (-0.35;0.27) |  | 0.17 | -0.06 (-0.37;0.26) |  |
|  | | | | | |  | | |  | | |
| **Stroke** | No stroke | 838 | -0.008 | Ref | \| 0.53 \| \| --- \| \|  \| | -0.004 | Ref | \| 0.40 \| \| --- \| \|  \| | -0.003 | Ref | \| 0.73 \| \| --- \| \|  \| |
|  | Stroke | 40 | 0.16 | 0.09 (-0.2;0.38) |  | 0.09 | 0.12 (-0.17;0.42) |  | 0.06 | 0.05 (-0.25;0.35) |  |
|  | | | | | |  | | |  | | |
| **Angina** | No angina | 685 | 0.03 | Ref | \| 0.17 \| \| --- \| \|  \| | 0.003 | Ref | \| 0.47 \| \| --- \| \|  \| | -0.02 | Ref | \| 0.06 \| \| --- \| \|  \| |
|  | Angina | 192 | -0.09 | -0.1 (-0.26;0.05) |  | 0.0001 | 0.06 (-0.1;0.21) |  | 0.08 | 0.15 (-0.01;0.30) |  |
|  | | | | | |  | | |  | | |
| **HIV** | No HIV | 322 | 0.03 | Ref | \| 0.67 \| \| --- \| \|  \| | -0.09 | Ref | \| 0.32 \| \| --- \| \|  \| | -0.02 | Ref | \| 0.26 \| \| --- \| \|  \| |
|  | HIV | 483 | 0.05 | 0.04 (-0.14;0.21) |  | 0.09 | -0.09 (-0.26;0.09) |  | 0.11 | -0.10 (-0.28;0.08) |  |
| 1. Missing recall data only for one participant 2. Missing digit span data only for one participant 3. Missing digit span and verbal fluency data for one participant 4. Higher scores represent better cognition 5. Linear regression controlled for sex, age group, residence, marital status, education, socioeconomic position, tobacco use, and BMI | | | | | | | | | | | |
