# Supplementary material for: Cognition in older adults in Uganda: Correlates, trends over time and association with mortality in prospective population study
Source: PLOS Glob Public Health. 2023 Nov 3;3(11):e0001798. doi: 10.1371/journal.pgph.0001798 (PMC10624290; doi:10.1371/journal.pgph.0001798)
Supplement: S3 Table — (DOCX) [file pgph.0001798.s003.docx]

S3 Table. Baseline characteristics stratified by whether participants completed the follow up period

|  |  | **Not lost to follow up (%)** | **Lost to follow up or died (%)** |
| --- | --- | --- | --- |
|  |  |  |  |
|  | **Total** | 378 | 131 |
|  |  |  |  |
| **Sex** | Male | 143 (38) | 55 (42) |
|  | Female | 235 (62) | 76 (58) |
|  |  |  |  |
| **Residence** | Rural | 224 (59) | 32 (24) |
|  | Urban | 154 (41) | 99 (76) |
|  |  |  |  |
| **Age group** | 50-59 | 136 (36) | 42 (32) |
|  | 60-69 | 105 (28) | 45 (34) |
|  | 70-79 | 94 (25) | 33 (25) |
|  | 80+ | 43 (11) | 11 (8) |
|  |  |  |  |
| **Educational attainment^1^** | No formal education | 98 (26) | 20 (15) |
|  | Any formal education | 279 (74) | 111 (85) |
|  |  |  |  |
| **Marital status** | Married/ cohabiting | 127 (34) | 38 (29) |
|  | Not married | 251 (66) | 93 (71) |
|  |  |  |  |
| **Socio-economic position** | 1 (Lowest) | 87 (24) | 31 (24) |
|  | 2 | 79 (21) | 31 (24) |
|  | 3 | 78 (21) | 20 (15) |
|  | 4 | 81 (22) | 24 (19) |
|  | 5 (Highest) | 46 (12) | 24 (19) |
|  |  |  |  |
| **Stroke** | No | 12 (3) | 4 (3) |
|  | Yes | 366 (97) | 127 (97) |
|  |  |  |  |
| **Angina** | No angina | 282 (75) | 96 (73) |
|  | Angina | 95 (25) | 35 (27) |
|  |  |  |  |
| **HIV** | HIV negative | 238 (63) | 72 (55) |
|  | HIV positive | 140 (37) | 59 (45) |
|  |  |  |  |
| **Cognition score groups** | 5 (highest) | 78 (21) | 24 (19) |
|  | 4 | 71 (19) | 29 (22) |
|  | 3 | 65 (17) | 26 (20) |
|  | 2 | 78 (21) | 25 (19) |
|  | 1 (lowest) | 86 (23) | 26 (20) |
| 1. Missing data (Educational attainment, angina, cognition score, CV disease: missing data for 1 participant; Socio-economic position: missing data for 8 participants) | | | |
