# Supplementary figures and images for: Cognition in older adults in Uganda: Correlates, trends over time and association with mortality in prospective population study
Source: PLOS Glob Public Health. 2023 Nov 3;3(11):e0001798. doi: 10.1371/journal.pgph.0001798 (PMC10624290; doi:10.1371/journal.pgph.0001798)

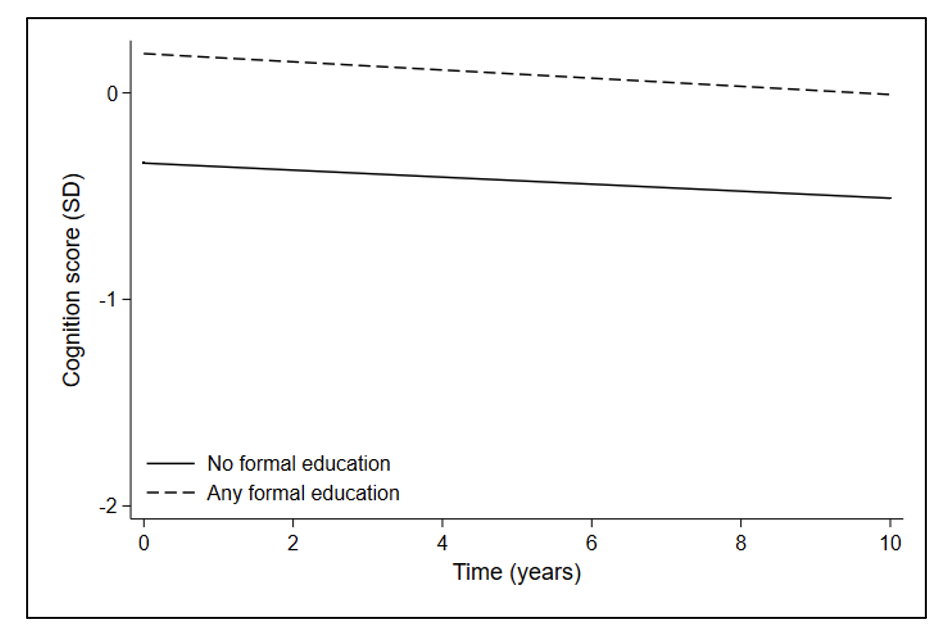

Supplement: S1 Fig — (TIFF) [file pgph.0001798.s005.tiff]

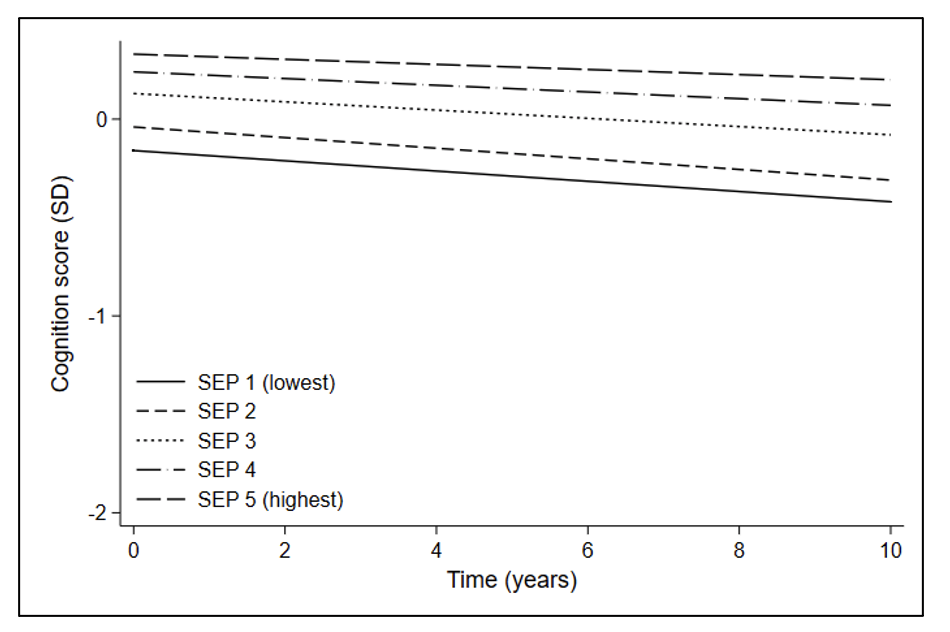

Supplement: S2 Fig — (TIFF) [file pgph.0001798.s006.tiff]
