# Supplementary material for: Cognition in older adults in Uganda: Correlates, trends over time and association with mortality in prospective population study
Source: PLOS Glob Public Health. 2023 Nov 3;3(11):e0001798. doi: 10.1371/journal.pgph.0001798 (PMC10624290; doi:10.1371/journal.pgph.0001798)
Supplement: Attachment — Submitted filename: Response to reviewers Plos global public health.docx [file pgph.0001798.s007.docx]

Response to reviewers Plos global public health

Journal requirements

| Comment | Response |
| --- | --- |
| Please include a complete copy of PLOS’ questionnaire on inclusivity in global research in your revised manuscript. | This form is now included |
| Please amend your detailed Financial Disclosure statement. This is published with the article. It must therefore be completed in full sentences and contain the exact wording you wish to be published. | This statement has been amended |
| Please provide separate figure files in .tif or .eps format. | These figures have now been provided |
| Deposition of data | We have clarified that The Wellbeing of Older People Study data are available through the WHO Multi-Country Studies Data Archive https://apps.who.int/healthinfo/systems/surveydata/index.php/catalog |

Reviewer comments

| Comment | Response |
| --- | --- |
| Reviewer #1: The study is interesting and relevant. It is not clear if the Wellbeing of Older People Study (WOPS) was conducted throughout the year or during specific times of the year. That way, they can determine weather conditions as influencing results as well. The next steps can also be to explore other factors such as the potential links between precipitation and age-related cognitive decline. And the role of diet in cognitive decline among such populations. | WOPS rounds were collected throughout the year until all study participants were covered. This is now specified in the methods.  As such, weather conditions are unlikely to have influenced the results. We agree that future paper assessing links of precipitation and diet and cognitive decline would be of interest. |
|  |  |
| Title - The paper is about the association of the changes in cognition with HIV or mortality or CVD -the independent variable is not specified-. Therefore, the title should change after reviewing all comments. | The paper is essentially descriptive – of the correlates of exposures with cognition and cognitive decline, and the relationship of cognition and mortality. We have adapted the title and short title accordingly. |
|  |  |
| Introduction - It is necessary to add quantitative data to support the statements, for example, the prevalence and incidence of dementia in Africa or the known risk factors (like HIV) for dementia, including their odds ratio values. | Quantitative data is added to support statements in two instances in the introduction, and estimates have been added on the prevalence of dementia. |
|  |  |
| The analysis contemplates certain variables not mentioned in the background, so there is any theoretical support for their inclusion in the article (disability, socioeconomic status, education). | The background has now been expanded to explain what mechanisms may link cognitive impairment and mortality, and hence provide rationale for the analysis plan. |
| The purpose of the article needs to be clarified. First, the dependent and independent variables must be identified. Also, the objective is poorly structured because it has two outcomes (dead and cognitive deterioration -no dementia-) and three dependent variables (CVD, HIV, disability). | We have now clarified that “The purpose of this study is to describe the correlates of baseline cognition (including sociodemographic, CVD, HIV, disability), trends in cognition over time, and association of cognition with mortality in a cohort of older Ugandan people.” |
| Material and Methods.  No inclusion, exclusion, or elimination criteria are mentioned. | Inclusion and exclusion criteria are now clarified in the methods. |
| The WHO cognition test needs to be sufficiently explained; the scores and assessment must be understood. Moreover, this test cannot diagnose dementia (just cognitive function), so it is impossible to analyze dementia risk factors. | More information was given on the verbal recall test.  The description of the scores is given on page 5 under outcomes.  We have rephrased to emphasise that we are not analysing dementia risk factors, but cognition risk factors. We have stated in the discussion that a key limitation is that there was no clinical diagnosis of dementia. |
| It has yet to explain how authors manage the changes down the cohort; for example, there is no information if the measurement of the variables from the first or the last wave or if the cognitive assessment was just for the ones from the first wave. Moreover, figures 2 and 3 (the title of figure 3 is wrong) do not specify if the values are from the follow-up observations from the first wave. | Cognition was measured at each wave – as now specified on page 4.  In Figure 2, the change in cognition over time was described. For tables 1, 2, 3, baseline cognition score is described in relation to other variables. This is now clarified in the text and tables and figures. “Baseline” means the first cognition score for the participant (often Wave 1, but sometimes a later Wave) which is specified in the methods.  The figure titles have now been corrected. |
| It is necessary to differentiate between the dependent and independent variables and the covariates. | The dependent, independent variables and covariates are now stated in the analysis section of the methods. |
| Also, a more accurate explanation of the socioeconomic position and educational attainment variable construction is required. | Socioeconomic position variable was developed using Principal Components analysis and this is now explained further in the methods.  There were only two categories for educational attainment, based on self-report from the questionnaire – no formal or any formal education, as stated in the methods. |
| The way the cognitive variable was treated is confusing (described on page 5); it seems arbitrary. Also, including participants that do not have all the components from the cognitive questionnaire and transforming the original disability score of 60 to 100 is methodologically incorrect. | We have stated in the limitations now that there was not a standardised way to create a cognitive variable. There were very few participants missing data, and so including them in the analyses would not have affected the results. The disability score out of 60 was transformed to be out of 100 to allow comparability with other studies. |
| A better redaction of the statistical analysis section is required, step by step.  The linear regression technique can only be used if the independent variable is explicit. Moreover, mortality cannot be estimated with Poisson analysis (page 6). | More information is given on the statistics, describing the independent and dependent variables. We have clarified that we used Poisson analysis to estimate mortality rates. |
|  |  |
| Results.  This section should be modified, taking into consideration the previous comments.  It needs to be clarified the way information is organized. Please, do not combine descriptive and association results (table 1, 2, 3). | The section has been modified to clarify when baseline cognition scores were used.  We would prefer to leave the tables in their current format, as we believe it provides a coherent flow and otherwise we would need to include many more tables. |
| On page 9, it needs to be clarified why the authors calculated the person-years value. | The authors calculated person years to allow mortality rates to be estimated and compared. |
| Additionally, talking about the crude mortality rate needs to be corrected (crude mortality is the mortality of the total population, not a sample). | Crude has been corrected to unadjusted. |
|  |  |
| Discussion.  This section should be modified, taking into consideration the previous comments. |  |
| The discussion is focused on the relationship between the findings and dementia. However, cognitive function decline could have a lot of reasons (like aging and traumas), so I see an author bias. | The discussion has been rephrased to focus on cognition rather than dementia. In the limitations we acknowledge that cognition is influenced by many factors. |
| In the limitations section, the authors said that one of the article's strengths is that with this data, they can analyze the differences between populations with and without HIV, which is not valid. Additionally, one of the limitations listed is that with the scale used, it is not possible to assess dementia or related risk factors, so -by knowing this from the beginning- there is a contradiction with the objective of the article. | We have rephrased to state that we can describe differences in cognition and cognitive decline in people with and without HIV.  The objective is now restated to focus on cognition, not dementia. |
| Conclusion.  This section should be reviewed, taking into consideration all the comments above. | Rephrased to state “The crucial next step will be to investigate types and presentation of clinical dementia in this cohort, so we can better understand the clinical relevance of these findings to inform public health planning.” |
| General comments.  Review English redaction. Review the correct definitions of acronyms. Figure 1 does not have a title. Figure 3 has the wrong title. | These corrections have been made |
