# Supplementary material for: Cognition in older adults in Uganda: Correlates, trends over time and association with mortality in prospective population study
Source: PLOS Glob Public Health. 2023 Nov 3;3(11):e0001798. doi: 10.1371/journal.pgph.0001798 (PMC10624290; doi:10.1371/journal.pgph.0001798)
Supplement: Attachment — Submitted filename: Second response to reviewers.docx [file pgph.0001798.s008.docx]

PGPH-D-23-00011R1
Cognition in older adults in Uganda: correlates, trends over time and association with mortality in prospective population study
PLOS Global Public Health

Journal Requirements:

1. Please include a complete copy of PLOS’ questionnaire on inclusivity in global research in your revised manuscript.

**Response: The questionnaire on inclusivity has been completed.**

2. **Comments to the Author**

   Reviewer #2: association with mortality in a prospective population study

   Introduction

   There is any theoretical support for including certain variables in the article (like disability, socioeconomic status, education, IALS, and ADLs). Also, a more extensive literature review is required to understand the problem addressed.

**Response: The introduction has been expanded to explain why the variables disability, socio-economic status, education, IADLS and ADLS were included.**

The aim still needs to be clarified. For example, the “cognitive score” seems to be the dependent variable, so what does “baseline cognition (including sociodemographic, CVD, HIV, disability)” means in the objective?

**Response: The aim has been clarified.**

Again, it needs to be clarified the objective of the article. In the limitation section, the authors said that one of the strengths is the possibility of comparing persons with and without HIV. However, the authors should have made this comparison in the analysis and methodology. It seems a contradiction.

**Response: We have clarified that we will compare trends in cognition over time including whether this varied by cardiovascular disease or HIV status.**

Methodology
No inclusion, exclusion, or elimination criteria are mentioned. A more specific description of the cohort is required. Lines 97-106 of page 26 need to give more information to understand how the cohort was constructed. Moreover, the aggregate classification (numbers 1 to 5) needs to be clarified.

**Response: More information is now provided on the cohorts, including the description of the 5 groups. The only inclusion/exclusion criteria was on the basis of residence, which is specified.**

If cognition was measured at each wave, extensive detail is required of how the “cognition score” was constructed considering the time measures of cognition in every wave (specifying what happened with the observations that do not have the 4 cohort measures).

**Response: One cognition score was calculated for participants at each wave, where data was available for that participant. The baseline cognition score was their first measure of cognition at cohort entry. This is now clarified in the methods.**

The outcome variable cannot be measured like that (cognition score). The arbitrary “combination” of the three different components of cognition is wrong (even if the authors specify it in the limitations). Indeed, it is not clear what this “cognition score” means. Again, including participants that do not have all the components from the cognitive questionnaire and transforming the original disability score of 60 to 100 is methodologically incorrect. So, authors cannot define low, middle, or high cognition. Consequently, results and conclusions do not have the support to be interpreted.

**Response: Combining cognition scores into one composite has been undertaken by a range of other studies.** **[1] Indeed, some argue that composite scores are more comprehensive and reliable than single scores.** **[2] Moreover, using these scales independently would raise concerns about the vast numbers of analyses/comparisons made and the complexity of the paper. There is, however, a lack of standard approaches to creating composites. [1] Weighting scales is a possibility, but there is not a clear rationale why one would have more weight than another. Consequently, we chose a simple and transparent approach where we calculated z-scores for each component and created an overall score of the mean of the 3 component scores. This approach is frequently taken in the literature. [1, 3].**

**We therefore maintain for these reasons that it was appropriate to combine the three scores into one overall cognition score. We have been transparent in the methods of how the cognition score was created, and have clarified in the limitations that this is not a standardised approach and that this raises concerns about its validity and interpretability.**

**The number of participants missing 1 or 2 components was very small, as stated in the methods, and so including those participants in the analyses would not have affected the overall results.**

**We have edited the paper to refer to cognition score, rather than cognition, throughout. We do not define low, middle or high cognition in the paper.**

**Supporting references:**

1.	Schneider, L.S. and T.E. Goldberg, *Composite cognitive and functional measures for early stage Alzheimer's disease trials.* Alzheimers Dement (Amst), 2020. **12**(1): p. e12017.

2.	Amaefule, C.O., et al., *Association between composite scores of domain-specific cognitive functions and regional patterns of atrophy and functional connectivity in the Alzheimer's disease spectrum.* Neuroimage Clin, 2021. **29**: p. 102533.

3.	Coley, N., et al., *Evaluating the clinical relevance of a cognitive composite outcome measure: An analysis of 1414 participants from the 5-year GuidAge Alzheimer's prevention trial.* Alzheimers Dement, 2016. **12**(12): p. 1216-1225.

Analysis
Poisson regression analysis cannot calculate mortality rates.

**Response: We have clarified that Poisson regression analysis was used to estimate the association of mortality rates and baseline cognition score, which is a commonly used statistical approach.**

The authors should have mentioned how they managed the loss of 26% of the sample.

**Response: We have stated in the methods that “Individuals contributed exposure time from the date of their first interview until the date of their last interview or death, whichever came first. Participants lost to follow-up were censored at a date halfway between their last interview date and the median date of data collection for the following wave.”**

Results
Separating descriptive and association results is better because it makes it simple to understand the findings. Therefore, leaving the descriptive and association results could be better.

**Response: The study was an observational cohort study. It is therefore not clear in our view whether there is a true distinction between descriptive and association/analytical comparisons.**

Again, why the authors calculated and included the person-years value must be clarified. The reason given by the authors needs to explain it.

**Response: Person-years were calculated as this was an open cohort and so other measures of incidence (e.g. risk) were not appropriate. The person years were used to allow estimation of mortality rates, which was used within the Poisson regression analysis. This is now clarified in the methods.**

Discussion
Results cannot be interpreted due to the lack of support for the “cognition score” because the variable does not measure cognition. So, all the statements about the findings are incorrect (lines 221-222-223; 232; 234-235; 246-247; 259-260).

**Response: We have adapted the discussion (and the rest of the paper) to emphasise that the study focused on cognition score, rather than cognition. Please see our argument above for the validity of the cognition score used.**

The strength list at the beginning of the limitations paragraph (page 33) must be corrected. The cohort needed more robust methodology support because it was the product of the fusion of diverse cohorts with different inclusion and exclusion criteria. Additionally, the authors said that it had a loss of follow-up of 26% of the sample.

**Response: We have rephrased by taking out the word “robust”. We have stated in the limitations that loss to follow-up was 26%, and now also that we combined data from different cohorts.**

This paper needs to provide correct and acceptable conclusions due to the methodology mistakes in the outcome variable and the inappropriate use of statistical analysis techniques.

**Response: We have adapted the conclusions to take account of the reviewer’s concerns.**
